# Machine Learning Analysis of Factors Influencing Pediatric Telehealth Visits During COVID-19: A State-Level Comparison Using 2021–22 National Survey of Children’s Health Data

**DOI:** 10.3390/healthcare12212170

**Published:** 2024-10-31

**Authors:** Yu-Sheng Lee, Junu Shrestha, Matthew Evan Sprong, Xueli Huang, Sushil Tuladhar, Michael Y. Chuang

**Affiliations:** 1 School of Integrated Sciences, Sustainability, and Public Health, College of Health, Science, and Technology, University of Illinois at Springfield, Springfield, IL 62703, USA; ylee317@uis.edu; 2 School of Public Management and Policy, College of Public Affairs and Education, University of Illinois at Springfield, Springfield, IL 62703, USA; mspro2@uis.edu; 3 Department of Computer Science, College of Health, Science, and Technology, University of Illinois at Springfield, Springfield, IL 62703, USA; xhuan20@uis.edu; 4 Weaver Consultants Group, Naperville, IL 60563, USA; stuladhar@wcgrp.com; 5 Department of Management Information Systems, College of Business and Management, University of Illinois Springfield, Springfield, IL 62703, USA; ychuang2@uis.edu

**Keywords:** NSCH, COVID-19, telehealth, video, telephone, social determinant of health, LASSO, machine learning

## Abstract

**Background/Objectives**: The COVID-19 pandemic reduced in-person pediatric visits in the United States by over 50%, while telehealth visits increased significantly. The national use of telehealth for children and the factors influencing their use have been rarely studied. This study aimed to investigate the prevalence of telehealth use during the COVID-19 pandemic and explore the potential factors linked to its use at the state level. **Methods**: A cross-sectional study of the National Survey of Children’s Health (2021–22) sponsored by the federal Maternal and Child Health Bureau was performed. We used the least absolute shrinkage and selection operator (LASSO) regression to predict telehealth use during the pandemic. A bar map showing the significant factors from the multivariable regression was created. **Results**: Of the 101,136 children, 15.25% reported using telehealth visits due to COVID-19, and 3.67% reported using telehealth visits due to other health reasons. The Northeast states showed the highest telehealth use due to COVID-19. In the Midwest and Southern states, children had a lower prevalence of telehealth visits due to other health reasons. The LASSO regressions demonstrated that telehealth visits were associated with age, insurance type, household income, usual source of pediatric preventive care, perceived child health, blood disorders, allergy, brain injury, seizure, ADHD, anxiety, depression, and special needs. **Conclusions**: This study demonstrated significant variability in the use of telehealth among states during the COVID-19 pandemic. Understanding who uses telehealth and why, as well as identifying access barriers, helps maximize telehealth potential and improve healthcare outcomes for all.

## 1. Introduction

Severe Acute Respiratory Syndrome Coronavirus 2 (SARS-CoV-2), commonly known as COVID-19, was first reported in December 2019 in China. Due to its rapid spread worldwide, it was declared a pandemic on 11 March 2020, by the World Health Organization [1]. This global outbreak directly and indirectly affected regular medical operations and healthcare [2]. As of 31 December 2021, there were more than 287 million confirmed cases worldwide, with 5.4 million people dying from this disease, which is considered a “once-in-a-century pathogen” [3,4]. During the COVID-19 pandemic, many non-urgent care visits, including primary care visits, had to be postponed or canceled even though healthcare facilities remained open [5,6,7,8]. This led to missed preventive care such as vaccinations, delayed diagnosis of new medical conditions like Type 1 diabetes, and inadequate management of chronic diseases, including obesity, depression, anxiety, and Attention-deficit/hyperactivity disorder (ADHD) [9]. A study found that outpatient, emergency department, and inpatient visits decreased significantly by 80.9%, 37.0%, and 30.2%, respectively, across all age groups [10]. In the United States (U.S.), in-person well and acute pediatric visits decreased by more than 50% in 2020 compared to 2019 [9,11]. Additionally, a Canadian study revealed a 58% reduction in pediatric emergency department visits nationwide during the COVID-19 pandemic [12].

However, some technological methods offered potential solutions to address the changes demonstrated in healthcare visits during the pandemic [13,14,15]. Telehealth, or telemedicine, has become a vital aspect of healthcare delivery. Studies showed that telehealth effectively collects and monitors real-time data, leading to improvements in the health state, well-being, self-management skills, lifestyle habits, and quality of life of patients with chronic disease [16]. Although communication difficulties (e.g., unable to communicate verbally in English) remain significant obstacles to telehealth visits [17], telehealth facilitates virtual communication with healthcare professionals, providing easy access to relevant advice and information. This interaction enhances shared knowledge and benefits patients and providers by improving their preparedness for consultations and decision-making [16]. This innovative approach not only streamlines the healthcare process but also contributes positively to clinical outcomes [18,19]. It also effectively reaches remote or underserved populations, ensuring equitable access to healthcare services [20,21]. Studies found that individuals with reliable internet access and adequate computer skills generally feel confident using telehealth. Additionally, the use of telehealth is influenced by people’s perception of their health and their need for additional support for their health conditions. Those who consider their health to be poor are more likely to utilize telehealth services. Further research also identified several characteristics associated with telehealth usage, including males and younger people, private insurance, and higher income. These individuals also experienced physical, mental, or emotional problems that limited their activity, and they tended to live in urban areas [22]. Furthermore, telehealth is a cost-effective option that has demonstrated improvements in therapeutic efficacy and quality of care, and it saves time and reduces travel costs [16]. The economic implications of chronic heart failure management are promising. A study found that telemonitoring for congestive heart failure can save costs, largely due to decreased hospital admissions and the efficacy of telehealth in disease management [19]. A recent survey also revealed that 94% of patients were satisfied with their telehealth visits [17,23]. 

During the COVID-19 pandemic, many recognized telehealth as a valuable tool for reducing the infection risks for both patients and healthcare providers [24]. A retrospective cohort study among more than four million participants in California found that telehealth usage increased substantially during the COVID-19 pandemic [10]. Another retrospective cohort study also conducted at California’s Stanford Children’s Health revealed that telehealth visits increased by 40% in 2020 compared to 2019 [11]. Telehealth has also proven to offer significant benefits across various medical fields, including cardiology. Tedeschi and colleagues revealed that telemonitoring for Left Ventricular Assist Device patients increased, proving safe and effective. In addition, the use of telehealth consultations (TCs) also accelerated significantly due to the COVID-19 pandemic. Prior to the pandemic, the adoption of TCs faced challenges such as a lack of familiarity with technology, regulatory and legal concerns, and reimbursement issues. However, many of these barriers diminished because of the pandemic [19]. 

Telemedicine originated in the 1970s, and meant “healing at a distance” [25]. Chung and Lee’s study further proposed the definition of distance healing into three categories: mHealth (the use of mobile or wireless technologies for healthcare service), dHealth (a wider range of smart and connected devices, including data mining and artificial intelligence, etc.), and eHealth (a broader scope than mHealth, primarily encompassing the use of information and communications technologies) [13]. Telehealth, by definition, refers to healthcare services involving all healthcare professions [26]. It can be considered as both mHealth and dHealth [13]. Based on the National Survey of Children’s Health data, this study defined “telehealth” as any healthcare visit by video or telephone [27].

While previous studies have examined telehealth use among specific populations, there is still limited research exploration and national information is still lacking. The findings did not represent the U.S. child population’s use of telehealth, and the factors affecting its use in children are rarely investigated. This study aimed to examine the prevalence of telehealth use during the COVID-19 pandemic and explore the potential factors associated with telehealth use at the state level.

## 2. Materials and Methods

### 2.1. Source of Data

This study utilized the 2021 and 2022 National Survey of Children’s Health (NSCH) database. The SAS dataset from the Child and Adolescent Health Measurement Initiative (CAHMI) was accessed from childhealthdata.org. The Data Resource Center for Child and Adolescent Health, supported by Cooperative Agreement U59MC27866 from the U.S. Department of Health and Human Services, Health Resources and Services Administration (HRSA), Maternal and Child Health Bureau (MCHB), collected comprehensive data on physical and mental health, access to quality health care, and various aspects of children’s family, neighborhood, school, and social contexts [27,28,29]. These data encompass children aged 0 to 17 across all 50 states and the District of Columbia.

The surveys are administered through both mail and web-based methods by the U.S. Census Bureau, including the prior version of the NSCH and a second survey that incorporates questions related to children with special needs: “National Survey of Children with Special Health Care Needs”. Additional information about the sampling and administration process, survey methodology, nonresponse bias analysis, and other pertinent information can be found on the survey’s website [28]. The survey results were adjusted to represent the population of noninstitutionalized children nationally and in each state by weighting for demographic factors such as age, sex, race, and household size to provide representative data for each state [30].

### 2.2. Participants

The NSCH is a public database that does not contain any personal identifiers. The 2021 NSCH survey took place between July 2021 and January 2022 [31], while the 2022 NSCH was conducted from July 2022 to January 2023. Households received a mailed invitation asking an adult knowledgeable about the child’s health and healthcare—typically a parent—to complete a brief screener questionnaire. This questionnaire asked participants to identify all children aged 0–17 living in the household. If there were children in the household, participants who chose to respond online would be instructed on how to access the survey [27]. 

After obtaining approval from the Institutional Review Board (IRB) at the primary author’s university (IRB waiver number 24-052), we downloaded the 2021 and 2022 NSCH datasets, including a total of 104,995 children who completed surveys by parents/caregivers. We excluded 3859 children due to a lack of information on telehealth use, leaving us with 101,136 children for the analyses. Supplementary Figure S1 shows the details of the study protocol for inclusion and exclusion criteria.

### 2.3. Study Variables

#### 2.3.1. Outcomes

Telehealth visits during the COVID-19 pandemic were assessed by asking parents whether their child had any healthcare visits by video or phone in the past 12 months and, if so, whether these visits were due to the coronavirus pandemic. The possible responses were (1) Had no telehealth visits, (2) Had telehealth visits but not due to COVID-19, or (3) Telehealth visits were due to COVID-19 [27]. For the purposes of this study, “had no telehealth visits” was considered as not having telehealth visits. Telehealth visits were then categorized as “telehealth visits due to other health reasons“ or “telehealth visits due to COVID-19”. The NSCH questions used by this study are tabulated in Supplementary Table S1.

#### 2.3.2. Covariates

The covariates (Supplementary Table S1) included in the analyses as potential factors to be regressed on telehealth visits were (1) demographics: age, sex, race/ethnicity, child born in the United States, parent’s highest education level, family structure of the child’s household, number of family members, household had difficulty covering basics like food or housing, residence in metropolitan areas, English is the primary language spoken at home and household income, (2) health behaviors: type of insurance, consistent health insurance coverage during the past 12 months, missed, delayed, or skipped preventive check-ups due to COVID pandemic, usual source of pediatric preventive care, personal doctor or nurse for child, perceived child health, (3) parents’ physical and mental health, and (4) health conditions: allergy to food, drug, or insect, asthma, blood disorders, brain injury, cerebral palsy, seizure, attention-deficit/hyperactivity disorder (ADHD), autism spectrum disorder (ASD), headache, Tourette syndrome, anxiety, depression, deafness or problems with hearing, blindness or problems with seeing, and child with special health care needs (CSHCN). The poverty level variable in 2021 had 18.78%, and in 2022 had 19.53% missing values, which were imputed by the U.S. Census Bureau data. The NSCH defines CSHCN as children who have or are at increased risk for chronic physical, developmental, behavioral, or emotional conditions that require more health and related services than other children [27].

### 2.4. Statistical Methods

The prevalence of telehealth visits was calculated using the SAS SURVEYFREQ procedure. The results represent the population of noninstitutionalized children nationally and in each state [27,30]. To identify predictors for telehealth visits during the COVID-19 pandemic, we employed a Least Absolute Shrinkage and Selection Operator (LASSO) model selection method. The LASSO method is designed to automatically select sparse models with nonzero coefficients for only a small fraction of the predictor. This approach is particularly useful when the number of predictors is large compared to the sample size, as it helps create models with fewer parameters, thereby reducing the risk of overfitting [32]. We used the R *glmnet* package to select variables via LASSO regression. When selecting optimal models, this study found that using the minimum cross-validation error often results in an overfit model. As a solution, Breiman and colleagues suggested using the maximum (i.e., one standard error rule) λ value to create a more parsimonious model [33]. 

Machine learning splits data randomly into the training set (70%) and validation set (30%) within the LASSO method to cross-validate the selected models. The prediction model selected from the training set was applied to the validation set to validate and evaluate the prediction efficacy. The receiver operating characteristic (ROC) curve and the area under the curve (AUC) were estimated to verify the discrimination performance in the training and validation sets. The optimal cutoff also estimated the sensitivity and specificity through Youden’s J Index. The Calibration plot with a binary fringe plot and nomogram was created by the R *rms* package with 1000 bootstrapping re-samples.

For the *p*-values of univariate models, we used the false discovery rate (FDR) method to adjust the *p*-values of the multiple comparisons. Factors that were found to be significant in the univariate models were included in the multivariable regression analyses. All analyses were performed in SAS package version 9.4 (SAS Institute Inc., Cary, NC, USA) and R package version 4.4.0 (R Core Team 2023. R: A Language and Environment for Statistical Computing. R Foundation for Statistical Computing, Vienna. https://www.R-project.org/ [accessed on 16 June 2024]). The maps were created using the ArcGIS 10.8.2 Online map package (Esri, Redlands, CA, USA) and MapChart (https://www.mapchart.net/index.html [accessed on 16 June 2024]).

## 3. Results

### 3.1. Prevalence of Telehealth Visits by States Between 2021 and 2022

Across the United States, it is estimated that 16,583 (15.25%; 95% Confidence Interval (CI) = [14.77–15.74%]) U.S. children and adolescents under 18 years old reported that they had telehealth visits due to the COVID-19, while 3797 (3.67%; 95% CI = [3.42–3.92%]) had telehealth visits due to other health reasons during the coronavirus pandemic. Telehealth visits due to COVID-19 varied widely by state, ranging from 7.0% to 31.9%. Children living in the District of Columbia reported the highest percentage of telehealth use (31.91%; 95% CI = [28.03–35.79%]), followed by the State of Massachusetts (27.18%; 95% CI = [24.25–30.12%]) and Maryland (25.66%; 95% CI = [25.54–28.79%]). On the other hand, Idaho (7.43%; 95% CI = [5.68–9.19%]), Wyoming (7.40%; 95% CI = [5.79–9.01%]), and North Dakota (6.99%; 95% CI = [5.26–8.73%]) had the three lowest prevalences of telehealth visits due to COVID-19 (Table 1). 

The prevalence of using telehealth due to other health reasons was lower and ranged from 2.3% to 10.4% between states and districts. (Table 1) The District of Columbia had the highest telehealth usage at 10.35% (95% CI = [7.16–13.54%]), followed by the states of Florida at 6.53% (95% CI = [4.74–8.32%]) and California at 5.70% (95% CI = [4.25–7.16%]). North Dakota (2.31%; 95% CI = [1.49–3.12%]), Idaho (2.27%; 95% CI = [1.43–3.10%]), and Oklahoma (2.26%; 95% CI = [1.49–3.04%]) reported the lowest prevalence of telehealth usage due to other health reasons.

### 3.2. Multivariable Logistic Regression Modeling by LASSO Method

We used univariate and multivariable logistic regressions to identify the predictors of children’s telehealth visits during the coronavirus pandemic. 

#### 3.2.1. Predictors of Telehealth Visits Due to COVID-19 

Factors associated with telehealth visits due to COVID-19 in univariate analysis (Supplementary Table S2) were then included in the LASSO regression. 

The LASSO analysis selected 17 variables for the final multivariable logistic regression model (Supplementary Figure S2). The maximum (i.e., one standard error rule) λ = 0.008425. Table 2 demonstrates the multivariable logistic regression of the factors associated with telehealth visits due to the COVID-19 pandemic. Specifically, compared to household income ≥400% FPL, children with a household income of 200–399%, 100–199%, and 0–99% FPL were less likely to use telehealth healthcare visits during the pandemic (OR = 0.68; 95% CI = [0.63–0.72], *p* < 0.0001, OR = 0.69; 95% CI = [0.63–0.76], *p* < 0.0001, and OR = 0.64; 95% CI = [0.57–0.72], *p* < 0.0001). Children who missed preventive care in the past 12 months due to the COVID-19 pandemic were more likely to use telehealth due to COVID-19 (OR = 1.25). Children not having the usual source of pediatric preventive care and not having a personal doctor or nurse for children were less likely to use telehealth visits (OR = 0.50; 95% CI = [0.46–0.55], *p* < 0.0001 and OR = 0.75; 95% CI = [0.70–0.81], *p* < 0.0001). The perception of children’s health was associated with telehealth visits. Poorer health perception demonstrated more telehealth use (OR = 1.26 in good health perception and OR = 1.98 in fair to poor health perception). Health conditions such as allergy (OR = 1.13), brain injury (OR = 1.35), seizure (OR = 1.80), ADHD (OR = 1.67), anxiety (OR = 2.17), and depression (OR = 1.78) were found to be associated with telehealth visits. Children who needed special health care were found to be more likely to use telehealth healthcare (OR = 3.04; 95% CI = [2.83–3.26], *p* < 0.0001). The ROC curve reveals that the resulting model has great discrimination (Supplementary Figure S3). 

#### 3.2.2. Predictors of Telehealth Visits Due to Other Health Reasons 

Supplementary Table S2 also demonstrated the factors associated with the telehealth visits related to other health reasons in univariate logistic regression analysis. 

Fifteen variables were selected by the LASSO method for the final multivariable logistic regression model with the minimum λ = 0.001157 (Supplementary Figure S2). Older children were less likely to use telehealth visits during the pandemic. The ORs were 0.68. Children who missed preventive care in the past 12 months due to the COVID-19 pandemic were less likely to use telehealth due to COVID-19 (OR = 0.70; 95% CI = [0.60–0.81], *p* < 0.0001). Children not having a usual source of pediatric preventive care were less likely to use telehealth visits (OR = 0.73, *p* = 0.0031). The perception of children’s health was associated with telehealth visits. Children with poorer health perception demonstrated more telehealth use (OR = 1.35 in good health perception and OR = 1.98 in fair to poor health perception). Health conditions such as blood disorder (OR = 2.30), anxiety (OR = 1.41), and depression (OR = 1.83) were found to be associated with telehealth visits. Children who needed special healthcare were found to be more likely to use telehealth (OR = 1.72; 95% CI = [1.44–2.06], *p* < 0.0001). The ROC curve reveals that the resulting model has great discrimination (Supplementary Figure S3).

The common predictors of telehealth visits for both reasons (due to COVID-19 vs. due to other health reasons) included missed preventive care due to COVID-19, the usual source of pediatric preventive care, perceived child health, anxiety, depression, and CSHCN (Table 2). 

### 3.3. Prevalence Map and Predictors for Telehealth Use

**Figure 1:**
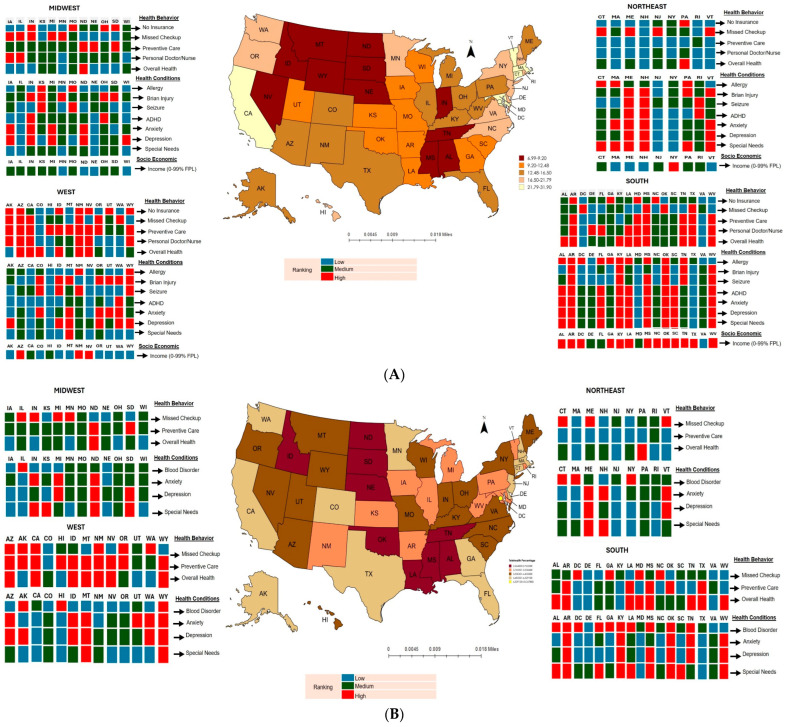
Prevalence map of telehealth use during COVID-19 pandemic. (**A**) Telehealth visits due to COVID-19. (**B**) Telehealth visits due to other health reasons.

Figure 1 combined different predictors to show potential associations between telehealth usage, health behaviors, and health conditions at the state level. Figure 1A illustrates the associations between telehealth visits due to COVID-19, health behaviors, health conditions, and household income at the state level. The Northeast showed the highest telehealth visits due to COVID-19. Conversely, the Midwest (Indiana, Nebraska, North Dakota, and South Dakota), South (Alabama, Mississippi, and Tennessee), and West (Idaho, Montana, Nevada, and Wyoming) had the lowest telehealth visit rates. These states generally fell into the highest tertile for not being insured, lacking a usual source of pediatric preventive care, not having a personal doctor or nurse for the child, reporting fair to poor health perception, having allergies to drug, food, or insect, or experiencing brain injuries, ADHD, anxiety, and depression. Apart from Midwestern states, most states generally had the highest tertile for low household income (0–99% FPL). This was especially evident in the southern states. Three states (Alabama, Mississippi, and Montana) had the highest percentage of CSHCN.

Figure 1B reveals a lower prevalence of telehealth visits during the pandemic due to other health reasons in the Midwest (Nebraska, North Dakota, and South Dakota) and Southern states (Alabama, Louisiana, Mississippi, Oklahoma, and Tennessee). These states, especially Louisiana, Mississippi, and Oklahoma, had the highest tertile of predictors, such as not having a usual source of pediatric preventive care, fair to poor health perception, anxiety, depression, and CSHCN (Figure 1B). However, Nebraska was an exception, with the lowest levels of all the predictors. One difference between the South and the Midwest was the prevalence of blood disorders (e.g., sickle cell disease) in children, with Southern states more likely to be in the highest tertile for blood disorders. On the other hand, nine states (Colorado, Delaware, Iowa, Missouri, New Jersey, Ohio, Rhode Island, Virginia, and Wisconsin) were in the lowest tertile for all predictors. Yet, they still had relatively higher telehealth visits during the pandemic, not related to the disease of COVID-19. Overall, children in the Northeast and West used more telehealth during the pandemic, unrelated to COVID-19.

## 4. Discussion

The COVID-19 pandemic significantly boosted the need for telehealth services. On average, monthly telehealth visits surged by over 2500% during the COVID-19 pandemic in 2020 compared to 2019 [15]. The significant rise in telehealth utilization has led to a transformation in healthcare delivery that is expected to endure even after the pandemic. This sets the stage for permanently incorporating pediatric telehealth services following the pandemic [15,34].

This large population-based repeated cross-sectional study, using the NSCH database, included 101,136 children aged 0 to 17 across all 50 states in the U.S. during the COVID-19 pandemic (2021–2022). We aimed to investigate the prevalence of telehealth use in US children during the COVID-19 pandemic and explore the potential factors associated with telehealth use at the state level. This study examined the use of telehealth because of (1) COVID-19 and (2) other health reasons during the pandemic. Of the 101,136 children, 16,583 (15.3%) reported using telehealth visits due to COVID-19, and 3797 (3.7%) reported using telehealth visits due to other health reasons, showing significant variation between states and districts. 

In the model of telehealth visits due to COVID-19, the LASSO regression found that telehealth usage was independently associated with insurance type, household income, missed preventive care due to COVID-19, usual source of pediatric preventive care, personal doctor or nurse for child, perceived child health, allergy, brain injury, seizure, ADHD, anxiety, depression, and CSHCN. On the other hand, the telehealth visits due to other health reasons model selected age, missed preventive care due to COVID-19, usual source of pediatric preventive care, perceived child health, blood disorders, anxiety, depression, and CSHCN as the predictors. This study, the first to investigate telehealth usage during the COVID-19 pandemic in U.S. children between 0 to 17 years, provides crucial insights and emphasizes the need for further research in pediatric telehealth visits. The LASSO regression is a shrinkage variable selection method that avoids overfitting problems in the analysis and overestimating of how well the model performs. It can also reduce the complexity of high-dimensional data. The authors deemed that LASSO regression is suitable for selecting predictors of telehealth usage [35,36,37,38,39].

Although the prevalence of telehealth visits varied across the states, we found geographic differences existed in the telehealth visits for both reasons (COVID-19 and other health reasons) during the COVID-19 pandemic. Our study, consistent with previous findings, [34,40] found that midwestern states (Nebraska, North Dakota, and South Dakota) and southern states (Alabama, Mississippi, and Tennessee) had the lowest telehealth usage percentages regardless of the reasons for visits. Although the univariate analyses found that children who lived in a metropolitan area were more likely to use telehealth for both reasons during the pandemic, the LASSO regression did not select this variable in the multivariable models when adjusted for other covariates. This finding was similar to the previous study [41,42]. However, another study conducted in Canada revealed that telehealth visits among rural patients increased from 11 to 147 per 1000 patients from 2012 to 2019, continuing until June 2020. Meanwhile, the growth in telehealth visits was even more significant for urban patients, rising from 7 to 220 visits per 1000 patients during the same period [43].

Accessing the quality of internet-based healthcare delivery is gradually regarded as a superdeterminant of health. It impacts healthcare outcomes more than traditional social determinants of health [44]. The digital divide refers to the gap between those with and without access to reliable broadband or high-speed internet [45]. The states of Nebraska, North Dakota, and South Dakota were in the geographic areas with rural broadband access below the national median or unreliable broadband access, while all three southern states (Alabama, Mississippi, and Tennessee) were considered in the geographic regions with rural broadband access below the national median [46,47,48]. According to the Federal Communications Commission and Broadband Now (https://broadbandnow.com/, a comparison and research website that does not offer internet [accessed on 15 August 2024]), 14.5 to 42 million people in the US lack access to reliable broadband service. This internet access discrepancy can significantly impact healthcare visits and health outcomes [46,49,50,51].

Limited broadband availability is associated with reduced use of telehealth services, largely due to financial limitations [51,52,53]. Household income was associated with the use of digital technologies. Wealthier families were more likely to adopt telehealth visits because of COVID-19 care during the pandemic [34,40,52]. This supported our findings, as the children reported a relatively lower household income (0–99% FPL) in the southern states than in other geographic regions, i.e., when they had COVID-19 and needed healthcare visits, they were less likely to use telehealth visits than children living in other states.

The lower broadband accessibility in the three midwestern states (Nebraska, North Dakota, and South Dakota) could be due to factors other than household income. These states have large rural areas with low population densities, making it economically challenging for service providers to establish broadband infrastructure due to high costs and a smaller potential customer base. Additionally, these states’ economies are primarily based on agriculture, which may not create as much demand for high-speed internet compared to more urban and industrialized regions, resulting in less investment in broadband infrastructure [54]. This lack of broadband infrastructure became evident during the pandemic, leading to a lack of access to telehealth visits via the Internet (note: connecting to telehealth appointments via phone can be an obstacle due to lower-quality reception). In 2021, the Infrastructure Investment Bill and American Jobs Act allocated USD 65 billion to improve broadband access and established the Affordable Connectivity Program (ACP). Under this initiative, eligible households can receive a USD 30 per month discount on their internet subscription and a one-time discount of up to USD 100 for purchasing a laptop, desktop computer, or tablet [51]. A survey by Education Superhighway (https://www.educationsuperhighway.org/, a nonprofit organization that connects classrooms with high-speed internet [accessed on 15 August 2024]) found that 75% of people were unaware of the program’s existence. For example, the survey found 277,968 eligible households in Idaho, 114,650 in North Dakota, and 129,617 in South Dakota, with enrollment rates of 9%, 7%, and 10%, respectively. These rates are lower compared to states with higher enrollment rates, such as Ohio (35%).

Compared to uninsured children, children with either public or private insurance tended to visit healthcare providers through telehealth because of COVID-19. When there was a reliable internet connection (e.g., in the states of Idaho and Nevada), the insurance status could play a role in these children’s healthcare experiences, necessitating a more nuanced approach to healthcare provision that considers the unique challenges [28]. Conversely, uninsured children in those internet unmet states (Alabama, Mississippi, Nebraska, North Dakota, and Tennessee) were not in the highest tertiles. In addition, insurance types were not associated with telehealth visits due to other health reasons. Our findings were similar to those of previous studies among adults. The study found that telemedicine visits were not associated with insurance coverage [34]. The Centers for Medicare and Medicaid Services (CMS) 1135 Waiver and Coronavirus Aid, Relief and Economic Security (CARES) Act have established several flexibilities and waivers to help healthcare providers during the COVID-19 pandemic [55,56,57]. Although the CARES Act and CMS 1135 Waiver did not restrict an existing relationship between patients and providers [56], in southern states like Alabama, Mississippi, and Tennessee, children without a personal doctor or nurse were less likely to use telehealth because of COVID-19. This was not the case in midwestern and western states such as Idaho, Montana, Nebraska, North Dakota, and South Dakota, where children generally had personal doctors or nurses and had fewer telehealth visits due to COVID-19. Physicians likely tended to use telehealth to treat patients with chronic diseases in fields such as endocrinology, rheumatology, gastroenterology, nephrology, and cardiology who were between 40 and 60 years of age [40,56].

During the pandemic, age was found to be associated with telehealth visits that were not related to COVID-19. Consistent with previous studies, younger children were more likely to use digital technologies than older ones [22,58]. Research also indicated that parents of children with disabilities use the internet more frequently compared to parents of healthy children. Studies have demonstrated that online support offers valuable practical and emotional assistance to parents, particularly when their child’s disability is uncommon [58,59,60]. The lockdown resulted in the cessation of in-person healthcare services for children with disabilities who were particularly vulnerable to the pandemic’s impact on healthcare delivery. These children may have varying clinical conditions. However, they all have common special healthcare needs that demand intensive interventions [61,62,63]. This explained the findings of our study that children with special healthcare needs care were more likely to use telehealth during the pandemic. However, there are many potential barriers to the use of telehealth on CSHCN. About 27% of parents reported feeling unprepared for and unsupported in the level of expected involvement in their child’s therapeutic services during the pandemic [64].

Regardless of the reasons for using telehealth visits, anxiety, depression, and CSHCN were found to be independently associated with telehealth use during the pandemic. Mental health was the most commonly concerned health condition for children who used telehealth visits during the COVID-19 pandemic [65,66]. Our findings are consistent with the previous studies. A study found a rapid increase in telehealth usage for mood and anxiety disorders (74.7%), psychosis (73.2%), substance use (83.6%), social problems (64.6%), and neurodevelopmental disorders (69.8%) from March 2020 through February 2021 [67]. This increase could be due to the increased incidences of mental health during the COVID-19 pandemic [68].

### 4.1. Study Limitations

This study has limitations as it did not explore household internet accessibility. Future studies should consider including this factor to better understand the reasons for children’s use of telehealth. This would lead to a more comprehensive understanding of the factors influencing telehealth usage. The questionnaire asked if the child had any healthcare visits by video or phone in the past 12 months, due to COVID-19 or due to other health reasons. The use of telehealth was only counted once in the dataset. However, it is possible that children used telehealth for other health reasons and later used it due to COVID-19 in the past 12 months. This may underestimate the prevalence of telehealth usage. In addition, we could not find enough information about the validation of NSCH questions regarding telehealth visits during COVID-19. Thus, interpreting the prevalences and its confidence intervals, one should be cautious. However, those questions were reported by parents. Parent-reported data mean that the surveys are completed by a parent, guardian, or other adult familiar with the child’s health. Although parent-reported data have been shown to overestimate child healthcare use [69], data from parents and caregivers are critical to fully understanding the broad spectrum of child health. Questions included in the survey instrument are deemed appropriate to be reported by parents. Data from parents who know their children better than anyone are critical to fully understand the broad spectrum of child health [70,71,72,73,74]. The findings of this study were based on the US child population; the generalization to other populations may be inappropriate. Lastly, this is a cross-sectional study; the causational inference of the association between predictors and telehealth use should be cautious.

### 4.2. Conclusions

The increased use of telehealth during the COVID-19 pandemic is expected to continue even after the pandemic ends. This trend presents an opportunity to revolutionize healthcare delivery. To ensure that telehealth remains a significant and beneficial part of healthcare, it is important to understand who is using the technology effectively and why, as well as to identify any barriers to access. By addressing these issues, we can maximize the potential of telehealth and improve healthcare outcomes for all.

## Figures and Tables

**Table 1:** Prevalence of telehealth visits during the COVID-19 pandemic by states between 2021 and 2022 (N = 101,136).

State/District	Telehealth Visits Due to COVID-19 (N = 16,583)			State/District	Telehealth Visits Due to Other Health Reasons (N = 3797)		
	Frequency ^1^	Percentage ^2^	95% Confidence Interval		Frequency ^1^	Percentage ^2^	95% Confidence Interval
North Dakota	100	6.99	(5.26, 8.73)	Oklahoma	48	2.26	(1.49, 3.04)
Wyoming	133	7.40	(5.79, 9.01)	Idaho	41	2.27	(1.43, 3.10)
Idaho	133	7.43	(5.68, 9.19)	North Dakota	45	2.31	(1.49, 3.12)
Mississippi	140	7.45	(5.84, 9.07)	Louisiana	44	2.32	(1.44, 3.20)
Montana	140	7.47	(5.81, 9.13)	Tennessee	70	2.34	(1.60, 3.09)
South Dakota	133	8.18	(6.39, 9.97)	Nebraska	54	2.53	(1.67, 3.39)
Alabama	149	8.22	(6.46, 9.99)	Mississippi	44	2.66	(1.65, 3.67)
Nebraska	204	8.59	(7.10, 10.08)	South Dakota	51	2.71	(1.80, 3.61)
Tennessee	239	8.67	(7.11, 10.23)	Alabama	48	2.77	(1.58, 3.95)
Nevada	156	9.20	(7.25, 11.16)	Arkansas	56	2.91	(1.93, 3.89)
Utah	189	10.21	(8.37, 12.05)	West Virginia	49	2.99	(1.94, 4.04)
Missouri	188	10.27	(8.31, 12.23)	New Mexico	62	3.13	(2.00, 4.27)
Indiana	206	11.07	(9.21, 12.94)	Iowa	55	3.14	(2.05, 4.24)
Georgia	338	11.34	(9.60, 13.08)	Illinois	46	3.14	(1.85, 4.44)
Arkansas	193	11.55	(9.37, 13.74)	Kansas	59	3.35	(2.31, 4.40)
Iowa	218	11.86	(9.94, 13.79)	Vermont	48	3.45	(2.24, 4.65)
Wisconsin	375	11.97	(10.17, 13.78)	Rhode Island	40	3.53	(2.09, 4.97)
Louisiana	184	11.99	(9.61, 14.36)	Missouri	51	3.60	(2.30, 4.90)
Oklahoma	204	12.01	(9.88, 14.14)	Ohio	101	3.64	(2.74, 4.55)
Kansas	220	12.35	(10.33, 14.37)	Wisconsin	88	3.67	(2.60, 4.74)
South Carolina	228	12.49	(10.34, 14.63)	New York	211	3.68	(2.89, 4.47)
Ohio	434	13.19	(11.68, 14.71)	Kentucky	47	3.75	(2.28, 5.21)
Florida	249	13.47	(11.33, 15.60)	North Carolina	68	3.75	(2.49, 5.01)
Illinois	271	13.95	(11.77, 16.14)	Hawaii	67	3.77	(2.46, 5.07)
Arizona	217	14.00	(11.45, 16.55)	South Carolina	48	3.79	(2.32, 5.25)
Michigan	247	14.22	(12.02, 16.41)	Montana	43	3.81	(2.26, 5.36)
Kentucky	234	14.22	(11.89, 16.54)	Nevada	60	3.88	(2.51, 5.25)
Texas	250	14.66	(12.15, 17.18)	Maine	55	3.92	(2.54, 5.30)
Alaska	279	14.68	(12.41, 16.96)	Michigan	64	3.92	(2.64, 5.20)
West Virginia	252	14.86	(12.47, 17.24)	Wyoming	92	4.02	(3.01, 5.03)
Colorado	542	16.01	(14.17, 17.86)	Indiana	59	4.15	(2.75, 5.55)
Pennsylvania	407	16.24	(14.21, 18.26)	Utah	76	4.16	(2.98, 5.35)
New Mexico	294	16.44	(13.90, 18.97)	Arizona	61	4.27	(2.77, 5.78)
Maine	312	16.51	(14.28, 18.73)	Pennsylvania	97	4.28	(3.05, 5.51)
New York	866	16.85	(14.77, 18.93)	Oregon	202	4.37	(3.48, 5.26)
Minnesota	279	16.95	(14.49, 19.40)	Virginia	63	4.40	(3.03, 5.77)
North Carolina	279	17.45	(14.74, 20.16)	Massachusetts	53	4.60	(2.86, 6.33)
Virginia	318	17.56	(15.00, 20.11)	Texas	70	4.68	(3.23, 6.13)
Washington	322	17.78	(15.25, 20.31)	Georgia	107	4.69	(3.51, 5.87)
Delaware	323	18.08	(15.56, 20.59)	Maryland	62	4.82	(3.10, 6.54)
Hawaii	411	18.84	(16.49, 21.19)	New Jersey	66	4.88	(3.34, 6.42)
Rhode Island	341	19.48	(16.69, 22.27)	Delaware	71	4.90	(3.30, 6.50)
New Jersey	337	20.40	(17.60, 23.21)	New Hampshire	48	4.92	(3.16, 6.69)
New Hampshire	343	20.53	(18.02, 23.05)	Alaska	62	5.04	(3.24, 6.84)
Oregon	1290	21.80	(20.22, 23.37)	Colorado	150	5.07	(3.95, 6.18)
Connecticut	388	23.29	(20.38, 26.20)	Connecticut	67	5.23	(3.59, 6.86)
California	1154	23.65	(21.24, 26.07)	Minnesota	73	5.27	(3.58, 6.97)
Vermont	414	24.61	(21.82, 27.39)	Washington	71	5.34	(3.56, 7.12)
Maryland	405	25.66	(22.54, 28.79)	California	307	5.70	(4.25, 7.16)
Massachusetts	438	27.18	(24.25, 30.12)	Florida	88	6.53	(4.74, 8.32)
District of Columbia	617	31.91	(28.03, 35.79)	District of Columbia	89	10.35	(7.16, 13.54)

^1^ Raw frequency of surveyed subjects with telehealth use due to COVID-19. ^2^ Weighted percentage of state pediatric population with telehealth use due to COVID-19.

**Table 2:** Multivariable logistic regression analysis for association of factors with telehealth visits.

	Telehealth Visits Due to COVID-19			Telehealth Visits Due to Other Health Reasons		
	OR ^1^	95% C.I. ^2^	*p*-Value ^3^	OR ^1^	95% C.I. ^2^	*p*-Value ^3^
Age						
<4	--	--	--	1		
4–8	--	--	--	0.81	0.65–1.01	0.1157
9–12	--	--	--	0.68	0.53–0.87	0.0080 *
13–17	--	--	--	0.68	0.54–0.86	0.0056 *
Parent’s highest education level						
Less than high school	1			1		
High school/GED	0.89	0.70–1.14	0.3590	0.78	0.50–1.22	0.3964
Greater than high school	1.23	0.98–1.55	0.0935	0.93	0.61–1.41	0.7635
Number of family members						
1 or 2	--	--	--	1		
3	--	--	--	1.03	0.77–1.38	0.8258
4	--	--	--	0.87	0.65–1.16	0.4735
5+	--	--	--	0.83	0.62–1.12	0.3601
Type of Insurance						
Uninsured	1			--	--	--
Public	1.45	1.19–1.76	0.0003 *	--	--	--
Private	1.55	1.29–1.88	<0.0001 *	--	--	--
Public + Private	2.06	1.65–2.57	<0.0001 *	--	--	--
Household income						
≥400% FPL ^4^	1			--	--	--
200–399% FPL	0.68	0.63–0.72	<0.0001 *	--	--	--
100–199% FPL	0.69	0.63–0.76	<0.0001 *	--	--	--
0–99% FPL	0.64	0.57–0.72	<0.0001 *	--	--	--
Missed preventive care in past 12 months due to the COVID-19 pandemic						
No	1			1		
Yes	1.25	1.18–1.33	<0.0001 *	0.70	0.60–0.81	<0.0001 *
Usual source of pediatric preventive care						
Yes	1			1		
No	0.50	0.46–0.55	<0.0001 *	0.73	0.61–0.87	0.0031 *
Personal doctor or nurse for child						
Yes	1			1		
No	0.75	0.70–0.81	<0.0001 *	1.06	0.91–1.24	0.5304
Perceived child health						
Excellent or very good	1			1		
Good	1.26	1.14–1.40	<0.0001 *	1.35	1.06–1.73	0.0360 *
Fair or poor	1.98	1.58–2.47	<0.0001 *	1.98	1.20–3.26	0.0213 *
Mother’s mental health						
Excellent or very good	--	--	--	1		
Good	--	--	--	0.97	0.83–1.14	0.7635
Fair or poor	--	--	--	0.92	0.69–1.22	0.6235
Child had the health conditions (Yes vs. No)						
Allergy to food, drug, or insect	1.13	1.06–1.21	0.0003 *	--	--	--
Asthma	--	--	--	1.26	0.99–1.60	0.1031
Blood disorders	--	--	--	2.30	1.18–4.49	0.0360 *
Brain injury	1.35	1.19–1.54	<0.0001 *	--	--	--
Cerebral palsy	1.43	0.94–2.17	0.1121	--	--	--
Seizure	1.80	1.32–2.46	0.0003 *	--	--	--
ADHD	1.67	1.53–1.82	<0.0001 *	1.12	0.88–1.41	0.4753
ASD	1.12	0.98–1.28	0.1166	1.33	0.96–1.85	0.1386
Headache	1.08	0.92–1.25	0.3590	--	--	--
Anxiety	2.17	1.99–2.37	<0.0001 *	1.41	1.11–1.80	0.0171 *
Depression	1.78	1.57–2.01	<0.0001 *	1.83	1.33–2.52	0.0015 *
Special health care needs (CSHCN)						
No	1			1		
Yes	3.04	2.83–3.26	<0.0001 *	1.72	1.44–2.06	<0.0001 *

^1^ OR = Odds Ratio. ^2^ C.I. = Confidence Interval. ^3^ False Discovery Rate *p*-value. ^4^ Federal Poverty Level. * *p*-value < 0.05.

## Data Availability

All data are provided within the manuscript.

## Supplementary Materials

The following supporting information can be downloaded at: https://www.mdpi.com/article/10.3390/healthcare12212170/s1, Figure S1. Flowchart of study inclusion/exclusion criteria. Table S1. A list of NSCH questionnaire questions was used in this study. Table S2. Univariate logistic regression analyses for association of factors with telehealth visits. Figure S2. Identification of the optimal penalization coefficient λ in the LASSO regression. Figure S3. ROC curves for the capability of predicting telehealth visits during COVID-19 and the calibration plots of the binary fringe plot with 1000 bootstrapping re-sample of LASSO regression for telehealth visits during COVID-19.
