# Supplementary material for: Machine Learning Analysis of Factors Influencing Pediatric Telehealth Visits During COVID-19: A State-Level Comparison Using 2021–22 National Survey of Children’s Health Data
Source: Healthcare (Basel). 2024 Oct 31;12(21):2170. doi: 10.3390/healthcare12212170 (PMC11545724; doi:10.3390/healthcare12212170)
Supplement: Supplementary file 1 [file healthcare-12-02170-s001.zip › healthcare-3222085-supplementary.pdf]

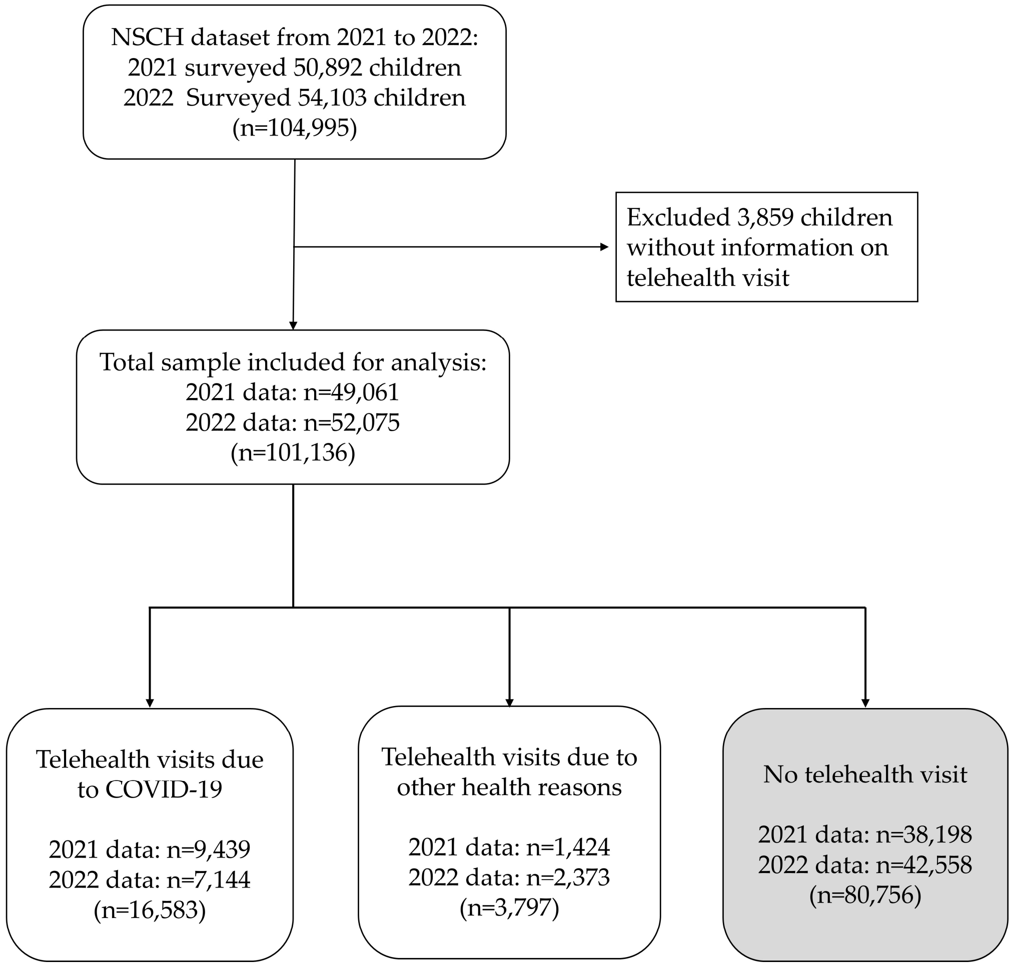


**Supplementary Figure S1.** Flowchart of study inclusion/exclusion criteria. Children who were excluded from the analysis are shown in rectangles. Children who reported no telehealth visits between 2021 and 2022 are shown in gray.

**Supplementary Table S1.** A list of NSCH questionnaire questions was used in this study.

| Variable | NSCH Questions |
|---|---|
| Telehealth visits | During the past 12 months, has this child had any health care visits by video or phone?<br>If yes, were any of this child's health care visits by video or phone because of the coronavirus pandemic? |
| Age | How old is this child? If the child is less than one month old, round age in months to 1. |
| Sex | What is this child's sex? |
| Race/Ethnicity | What is this child's race/ethnicity? |
| Child born in the United States | Was this child born in the United States? |
| Parent's highest education level | What is the highest education of adult in this child's household? |
| Family structure of child's household | What is the family structure that this child lives in? |
| Number of family members | How many of the people living or staying in the child's household are family members? |
| Residence in metropolitan area | The NSCH reported four geographic variables in the public use file: State of Residence, Core-Based Statistical Area Status, Metropolitan Statistical Area Status, and Metropolitan Principal City Status. |
| English is the primary language spoken at home | What is the primary language spoken in this child's home? |
| Type of Insurance | What type of health insurance coverage, if any, did the child have at the time of the survey? |
| Consistent health insurance coverage during the past 12 months | Did this child have consistent health insurance coverage during the past 12 months? |
| Household income | The following question is about your income. Think about your total combined family income in the last calendar year for all members of the family. What is that amount before taxes? *Include money from jobs, child support, social security, retirement income, unemployment payments, public assistance, and so forth. Also, include income from interest, dividends, net income from businesses, farm or rent, and any other money income received.*<br>This was then converted to the federal poverty level in the dataset. |
| Missed preventive care in past 12 months due to the COVID-19 pandemic | During the past 12 months, did this child miss, delay or skip any preventive check-ups because of the coronavirus pandemic? |
| Usual source of pediatric preventive care | During the past 12 months, how many times did this child visit a doctor, nurse, or other health care professional to receive a preventive check-up? (A preventive check-up is when this child was not sick or injured, such as an annual or sports physical, or well-child visit). |
| Perceived child health | In general, how would you describe this child's health? |
| Parents' physical health | What is the father/mother's general physical health status? |
| Parents' mental health | What is the general status of the father/mother's mental and emotional health? |
| Child had the health conditions | Has a doctor or other health care provider EVER told you that this child has the following health conditions? |
| Special health care needs (CSHCN) | Does this child have special health care needs based on the CSHCN Screener? |

**Supplementary Table S2.** Univariate logistic regression analyses for association of factors with telehealth visits.

| | Telehealth Visits Due to COVID-19 | | | Telehealth Visits Due to Other Health Reasons | | |
|---|---|---|---|---|---|---|
| Variables | **OR[1]** | **95% C.I.[2]** | **p-value[3]** | **OR[1]** | **95% C.I.[2]** | **p-value[3]** |
| **Age** | | | | | | |
| <4 | 1 | | | 1 | | |
| 4-8 | 0.89 | 0.84-0.95 | 0.0001* | 0.74 | 0.66-0.82 | <.0001* |
| 9-12 | 1.14 | 1.07-1.21 | <.0001* | 0.76 | 0.67-0.85 | <.0001* |
| 13-17 | 1.42 | 1.34-1.50 | <.0001* | 0.91 | 0.82-1.01 | 0.1110 |
| **Sex** | | | | | | |
| Female | 1 | | | 1 | | |
| Male | 0.99 | 0.95-1.03 | 0.4574 | 0.95 | 0.88-1.03 | 0.2925 |
| **Race/Ethnicity** | | | | | | |
| Black, non-Hispanic | 1 | | | 1 | | |
| Hispanic | 1.25 | 1.13-1.38 | <.0001* | 1.07 | 0.89-1.29 | 0.4674 |
| White, non-Hispanic | 1.17 | 1.07-1.27 | 0.0008* | 0.94 | 0.80-1.10 | 0.4427 |
| Other/Multi-racial, non-Hispanic | 1.20 | 1.08-1.32 | 0.0005* | 1.02 | 0.84-1.22 | 0.8931 |
| **Child born in the United States** | | | | | | |
| No | 1 | | | 1 | | |
| Yes | 1.09 | 0.96-1.23 | 0.1792 | 0.90 | 0.73-1.12 | 0.3880 |
| **Parent's highest education level** | | | | | | |
| Less than high school | 1 | | | 1 | | |
| High school/GED | 1.34 | 1.13-1.59 | 0.0009* | 1.20 | 0.89-1.63 | 0.2925 |
| Greater than high school | 2.08 | 1.77-2.44 | <.0001* | 1.51 | 1.14-2.00 | 0.0097* |
| **Family structure of child's household** | | | | | | |
| Two parents, currently married | 1 | | | 1 | | |
| Two parents, not currently married | 0.92 | 0.84-1.00 | 0.0651 | 0.92 | 0.77-1.09 | 0.3777 |
| Single parent | 1.00 | 0.95-1.06 | 0.9081 | 0.93 | 0.84-1.03 | 0.2552 |
| Grandparent household | 0.86 | 0.75-0.98 | 0.0212* | 0.79 | 0.61-1.03 | 0.1266 |
| Other | 1.48 | 1.22-1.78 | <.0001* | 1.41 | 0.98-2.03 | 0.1107 |
| **Number of family members** | | | | | | |
| 1 or 2 | 1 | | | 1 | | |
| 3 | 0.94 | 0.87-1.02 | 0.1677 | 1.09 | 0.92-1.28 | 0.3777 |
| 4 | 0.83 | 0.76-0.90 | <.0001* | 0.91 | 0.78-1.08 | 0.3444 |
| 5+ | 0.69 | 0.63-0.75 | <.0001* | 0.79 | 0.66-0.94 | 0.0149* |
| **Difficulty covering basics** | | | | | | |
| Never or rarely | 1 | | | 1 | | |
| Somewhat or very often | 1.26 | 1.18-1.34 | <.0001* | 1.05 | 0.93-1.19 | 0.4497 |
| **Residence in metropolitan area** | | | | | | |
| No | 1 | | | 1 | | |
| Yes | 1.69 | 1.59-1.80 | <.0001* | 1.36 | 1.21-1.52 | <.0001* |
| **English is the primary language spoken at home** | | | | | | |
| No | 1 | | | 1 | | |
| Yes | 1.35 | 1.24-1.46 | <.0001* | 0.92 | 0.80-1.05 | 0.2925 |
| **Type of Insurance** | | | | | | |
| Uninsured | 1 | | | 1 | | |
| Public | 2.38 | 2.06-2.74 | <.0001* | 1.33 | 1.06-1.67 | 0.0259* |

| | | | | | | |
|---|---|---|---|---|---|---|
| Private | 2.48 | 2.15-2.84 | <.0001* | 1.34 | 1.08-1.66 | 0.0172* |
| Public + Private | 3.80 | 3.24-4.47 | <.0001* | 1.78 | 1.36-2.34 | <.0001* |
| **Consistent health insurance coverage during the past 12 months** | | | | | | |
| No | 1 | | | 1 | | |
| Yes | 1.97 | 1.75-2.21 | <.0001* | 1.31 | 1.08-1.59 | 0.0138* |
| **Household income** | | | | | | |
| ≥400% FPL[4] | 1 | | | 1 | | |
| 200-399% FPL | 0.69 | 0.66-0.73 | <.0001* | 0.81 | 0.74-0.89 | 0.0319* |
| 100-199% FPL | 0.72 | 0.68-0.76 | <.0001* | 0.80 | 0.71-0.90 | 0.0003* |
| 0-99% FPL | 0.68 | 0.64-0.73 | <.0001* | 0.86 | 0.76-0.97 | <.0001* |
| **Missed preventive care in past 12 months due to the COVID-19 pandemic** | | | | | | |
| No | 1 | | | 1 | | |
| Yes | 1.49 | 1.40-1.58 | <.0001* | 0.73 | 0.63-0.86 | 0.0003* |
| **Usual source of pediatric preventive care** | | | | | | |
| Yes | 1 | | | 1 | | |
| No | 0.40 | 0.38-0.43 | <.0001* | 0.57 | 0.51-0.64 | <.0001* |
| **Personal doctor or nurse for child** | 1 | | | | | |
| Yes | 0.56 | 0.53-0.59 | <.0001* | 0.83 | 0.76-0.91 | 0.0003* |
| No | | | | | | |
| **Perceived child health** | | | | | | |
| Excellent or very good | 1 | | | 1 | | |
| Good | 2.59 | 2.42-2.76 | <.0001* | 2.05 | 1.80-2.33 | <.0001* |
| Fair or poor | 4.91 | 4.25-5.67 | <.0001* | 3.55 | 2.71-4.65 | <.0001* |
| **Mother's physical health** | | | | | | |
| Excellent or very good | 1 | | | 1 | | |
| Good | 1.21 | 1.15-1.27 | <.0001* | 1.07 | 0.98-1.18 | 0.2116 |
| Fair or poor | 1.47 | 1.34-1.60 | <.0001* | 1.20 | 1.01-1.43 | 0.0696 |
| **Father's physical health** | | | | | | |
| Excellent or very good | 1 | | | 1 | | |
| Good | 1.16 | 1.10-1.22 | <.0001* | 1.05 | 0.95-1.17 | 0.3712 |
| Fair or poor | 1.34 | 1.21-1.48 | <.0001* | 0.99 | 0.80-1.22 | 0.9105 |
| **Mother's mental health** | | | | | | |
| Excellent or very good | 1 | | | 1 | | |
| Good | 1.34 | 1.28-1.40 | <.0001* | 1.05 | 0.96-1.16 | 0.3542 |
| Fair or poor | 1.61 | 1.49-1.73 | <.0001* | 1.21 | 1.04-1.41 | 0.0261* |
| **Father's mental health** | | | | | | |
| Excellent or very good | 1 | | | 1 | | |
| Good | 1.29 | 1.22-1.36 | <.0001* | 1.07 | 0.96-1.19 | 0.2925 |
| Fair or poor | 1.75 | 1.59-1.92 | <.0001* | 1.13 | 0.93-1.39 | 0.2925 |
| **Child had the health conditions (Yes vs. No)** | | | | | | |
| Allergy to food, drug, or insect | 1.86 | 1.78-1.95 | <.0001* | 1.41 | 1.29-1.54 | <.0001* |
| Asthma | 2.23 | 2.08-2.39 | <.0001* | 1.69 | 1.47-1.95 | <.0001* |
| Blood disorders | 1.95 | 1.52-2.50 | <.0001* | 2.31 | 1.50-3.56 | 0.0004* |
| Brain injury | 2.09 | 1.91-2.29 | <.0001* | 1.77 | 1.48-2.12 | <.0001* |
| Cerebral palsy | 6.54 | 4.88-8.78 | <.0001* | 2.02 | 0.98-4.17 | 0.0965 |
| Seizure | 6.32 | 5.15-7.75 | <.0001* | 4.01 | 2.73-5.90 | <.0001* |

| | | | | | | |
|---|---|---|---|---|---|---|
| Attention-deficit/hyperactivity disorder (ADHD) | 4.22 | 3.98-4.46 | <.0001* | 2.56 | 2.27-2.88 | <.0001* |
| Autism Spectrum Disorder (ASD) | 3.83 | 3.50-4.20 | <.0001* | 2.24 | 1.84-2.73 | <.0001* |
| Headache | 3.03 | 2.73-3.37 | <.0001* | 2.21 | 1.78-2.74 | <.0001* |
| Tourette Syndrome | 5.79 | 4.28-7.85 | <.0001* | 3.13 | 1.66-5.89 | 0.0010* |
| Anxiety | 5.81 | 5.50-6.14 | <.0001* | 3.40 | 3.05-3.80 | <.0001* |
| Depression | 6.78 | 6.28-7.33 | <.0001* | 4.78 | 4.13-5.53 | <.0001* |
| Deafness or problems with hearing | 1.99 | 1.69-2.34 | <.0001* | 1.43 | 1.01-2.02 | 0.0807 |
| Blindness or problems with seeing | 1.81 | 1.56-2.10 | <.0001* | 1.21 | 0.87-1.68 | 0.3260 |
| **Special health care needs (CSHCN)** | | | | | | |
| No | 1 | | | 1 | | |
| Yes | 5.11 | 4.90-5.34 | <.0001* | 2.83 | 2.60-3.07 | <.0001* |

[1]OR = Odds Ratio.
[2]C.I. = Confidence Interval.
[3]False Discovery Rate p-value.
[4]Federal Poverty Level.
*p-value < 0.05.

(A)

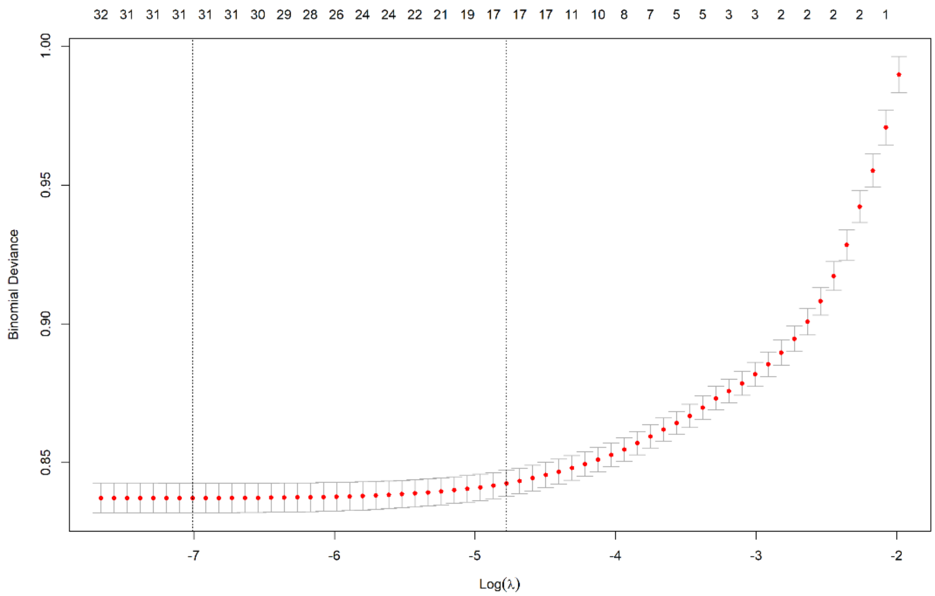
32 31 31 31 31 31 30 29 28 26 24 24 22 21 19 17 17 17 11 10 8 7 5 5 3 3 2 2 2 2 1
Binomial Deviance
1.00
0.95
0.90
0.85
-7
-6
-5
-4
-3
-2
Log(λ)

(B)

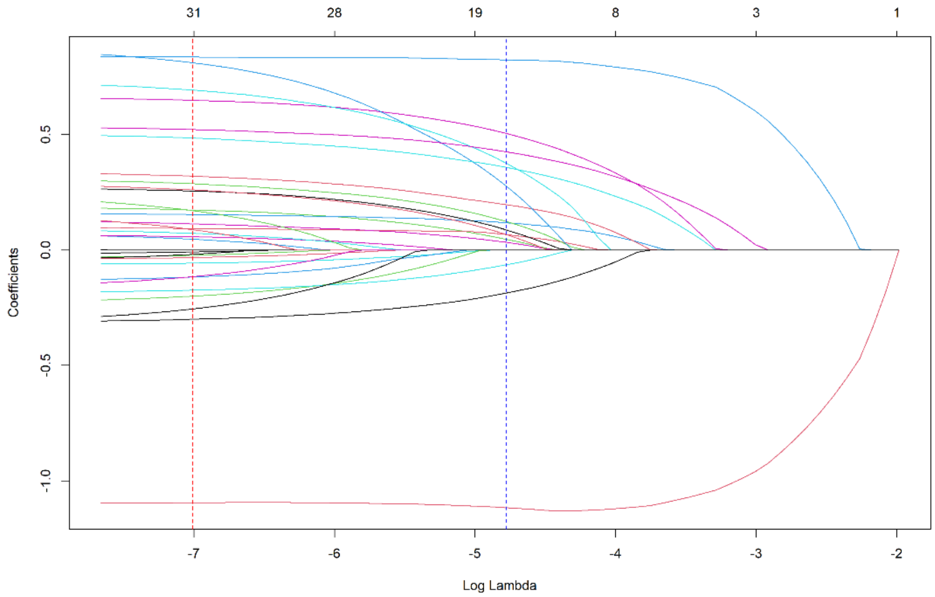
31 28 19 8 3 1
Coefficients
0.5
0.0
-0.5
-1.0
-7
-6
-5
-4
-3
-2
Log Lambda

(C)

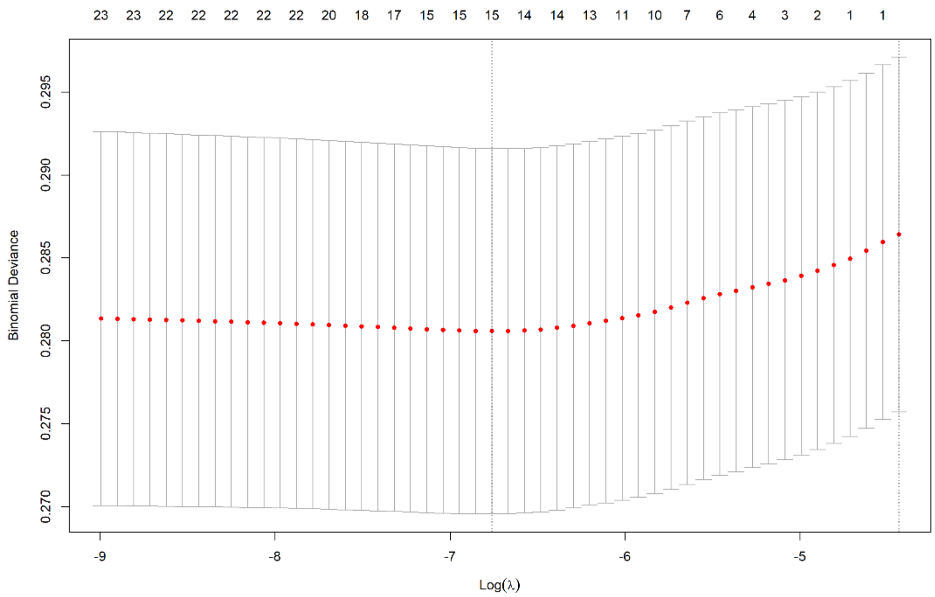


(D)

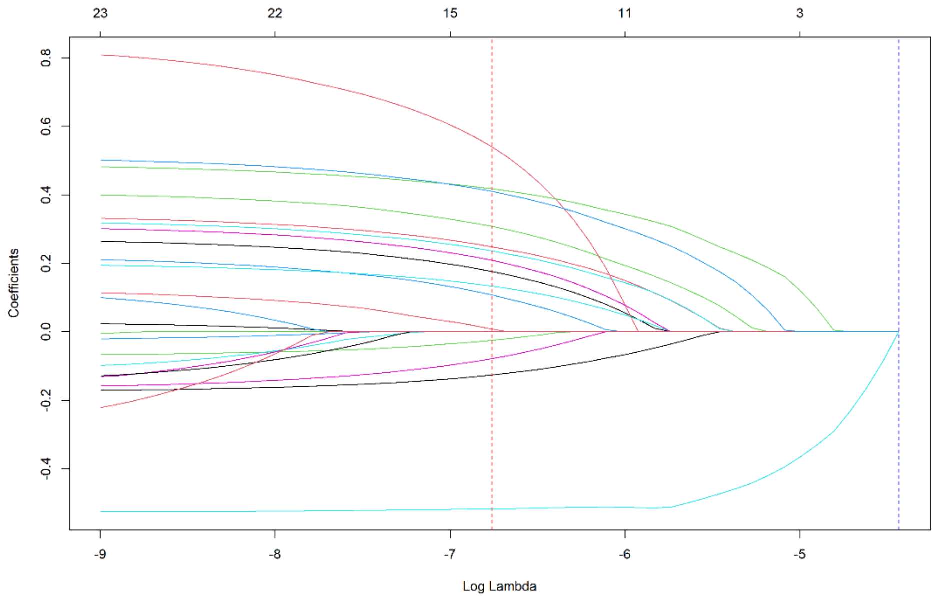


**Supplementary Figure S2.** Identification of the optimal penalization coefficient $\lambda$ in the LASSO regression. (A) and (B) are telehealth visits due to COVID-19; (C) and (D) are telehealth visits due to other health reasons.

(A) Training set

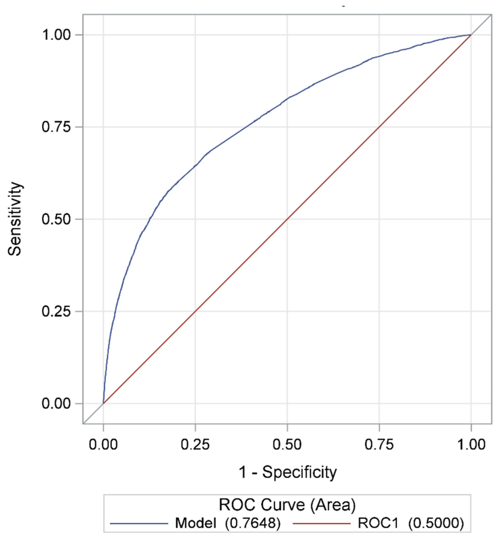


(B) Validation set

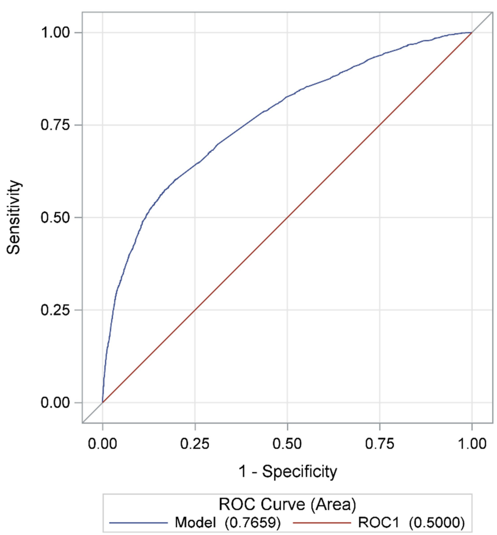


(C) Training set

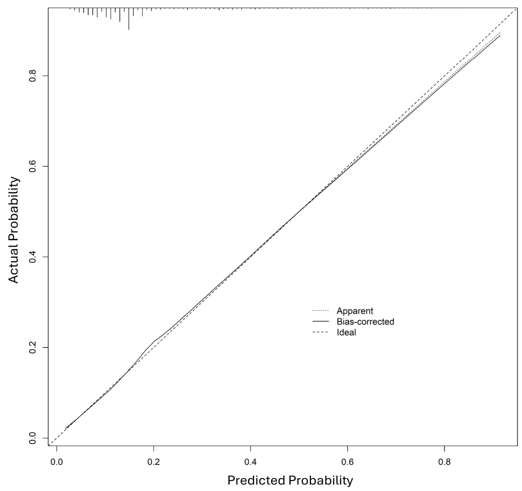


(D) Validation set

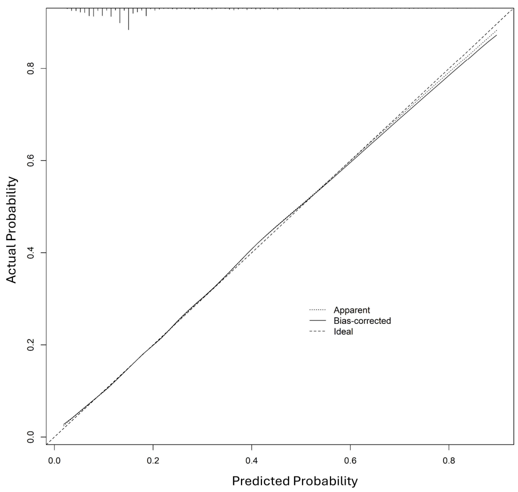

(E) Training set

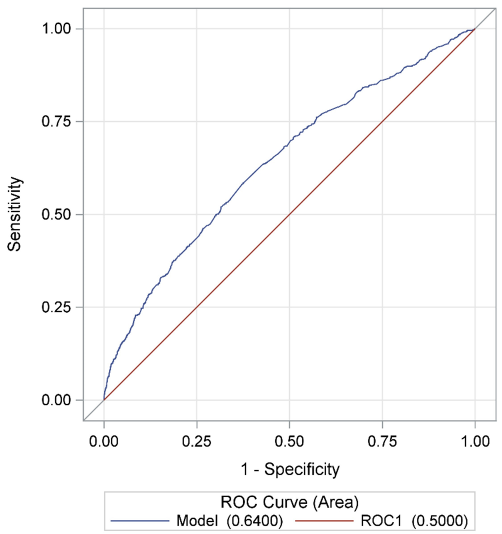


(F) Validation set

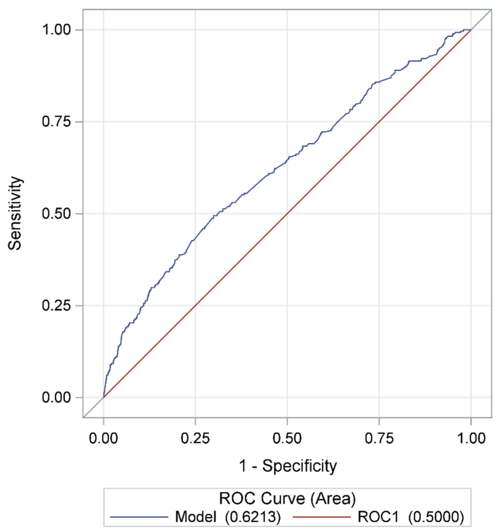


(G) Training set

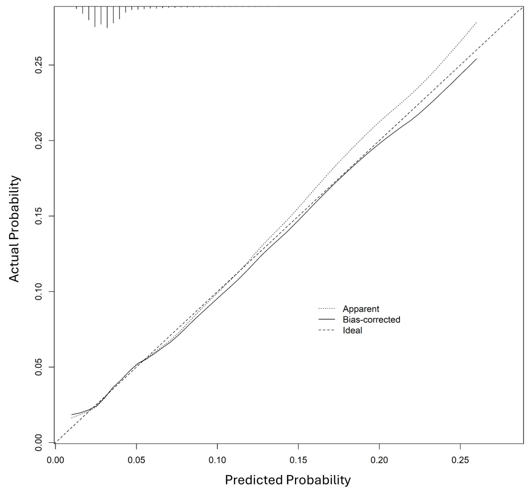


(H) Validation set

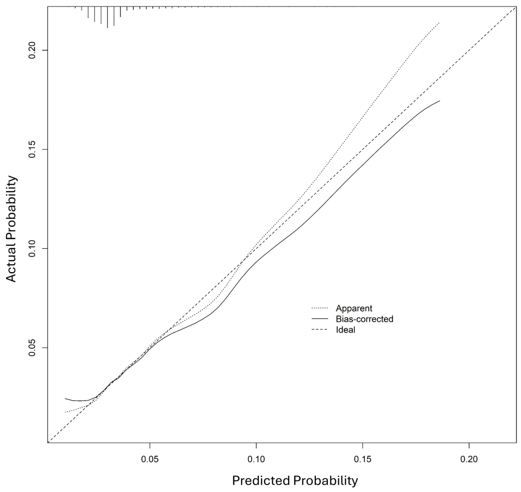


**Supplementary Figure S3.** (A) and (B) are ROC curves illustrating the capability in predicting the use of telehealth for telehealth visits due to COVID-19. The resulting model has great discrimination with an area under the curve (AUC) of 0.765 (95% CI: 0.757 - 0.772) in (A). The validation set also shows great discrimination in LASSO regression, with 0.766 (95% CI: 0.755 - 0.777) AUC. (C) and (D) are the calibration plots of the binary fringe plot with 1000 bootstrapping re-sample of LASSO regression for telehealth visits due to COVID-19. A curve that falls below the 45-degree line means underestimating the probability of the outcome. If the curve is above the line, the model may be overfitting. The calibration plots of models were used to provide better information about the selected models, graphically showing good agreement between the predicted and observed data in the training and validation cohorts. (E) and (F) are ROC curves illustrating the capability in predicting the use of telehealth for telehealth visits due to other health reasons. The LASSO regression training set to predict telehealth usage not related to COVID-19 showed good discrimination with an area under the curve (AUC) of 0.640 (95% CI: 0.618 - 0.662) in (E). The validation set also shows good discrimination in LASSO regression, with 0.621 (95% CI: 0.586 - 0.657) AUC. (G) and (H) are the calibration plots of the binary fringe plot with 1000 bootstrapping re-sample of LASSO regression for telehealth visits due to other health reasons.

The calibration plots of models were used to provide better information about the selected models, graphically showing good agreement between the predicted and observed data in the training and validation cohorts.
